# Place Identity: How Far Have We Come in Exploring Its Meanings?

**DOI:** 10.3389/fpsyg.2020.00294

**Published:** 2020-03-10

**Authors:** Jianchao Peng, Dirk Strijker, Qun Wu

**Affiliations:** ^1^College of Public Administration, Nanjing Agricultural University, Nanjing, China; ^2^Faculty of Spatial Sciences, University of Groningen, Groningen, Netherlands

**Keywords:** place identity, meanings, CiteSpace, scientometric, review

## Abstract

In order to synthesize the extensively studied place identities and their meanings, this paper reviews how researchers have conceived and deconstructed place identity. CiteSpace, a scientometric tool for visualizing and analyzing trends and patterns in scientific literature, is used to identify the active topics and new developments of publications in place identity. The data set input into CiteSpace consists of 1,011 bibliographic records retrieved from the core database of Web of Science with a title search of the articles published between 1985 and July 2019. The scientometric review reveals the extensive applications of place identity in various topics. Studies in this field experienced an active exploration in plural disciplines after 2000, and the hot area gradually concentrated on the discipline of humanities and social sciences after 2010 and shifted toward place marketing until now. A network of co-cited references identified seven dominant research clusters, of which the research on the influence of place identity on social actors’ attitudes and behaviors is most prominent and the research on the effects of physical environment change on place identity captures the latest emerging area. Versatile meanings of place identity are witnessed in different clusters and articles of a cluster. These meanings are intertwined in shaping the knowledge base of thematic concentrations. To supplement the scientometric analysis, a deep survey on measuring methods and roles of place identity in the contents of academic articles was done to trace knowledge connections between different empirical understandings of place identity. Finally, this paper summarizes the meanings of place identity in four dimensions and in turn offers some suggestions for further research directions.

## Introduction

Place identity is a versatile concept upon which many psychological theories of human–environment relations are built (Zimmerbauer et al., 2012; Gieseking et al., 2014). The social constructivist theory of place identity sheds light on individuals’ subjective perceptions of geographical space, providing valuable insights for studies of various disciplines, such as geography, sociology, psychology, environmental sciences and ecology, public administration, spatial planning, and so on (Haartsen et al., 2000). As broadly acknowledged, place identity was initially introduced by Proshansky (1978), who defined place identity as “those dimensions of self that define the individual’s personal identity in relation to the physical environment by means of a complex pattern of conscious and unconscious ideas, feelings, values, goals, preferences, skills, and behavioral tendencies relevant to a specific environment” (Proshansky, 1978, p. 155). Proshansky’s view on place identity has been widely referred to. Another dominant explanation of place identity can be found in most of Paasi’s articles (Paasi, 1986, 1991, 2002c, 2003, 2009a,b). He thought it would be beneficial to distinguish analytically two aspects of place identity, namely, place identity of a place and people’s place identity. The former refers to those features of nature, culture, and people that are used in the discourses and classifications of science, politics, cultural activism, regional marketing, tourism, governance, and political or religious regionalization, to distinguish one place from others. The latter refers to the identification of individuals with a place. The definitions of place identity by early leading scholars, such as Proshansky and Paasi, from different disciplines have influenced the formation of the versatile meanings of place identity to a great extent.

**FIGURE 1:**
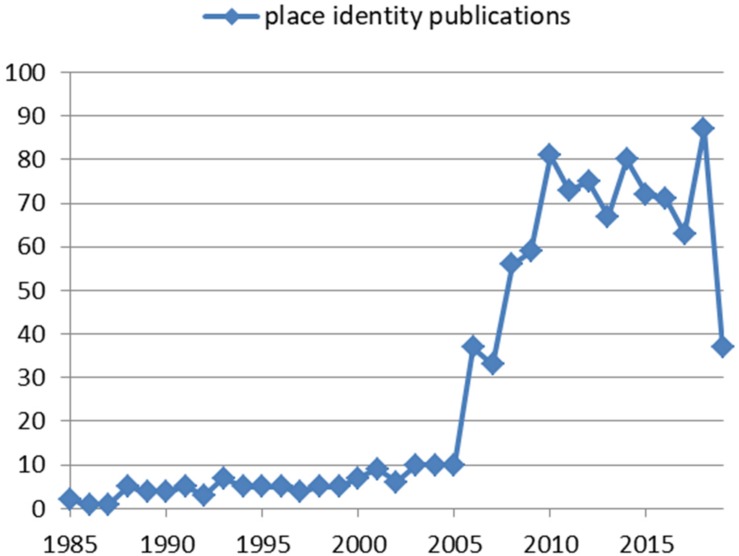
Number of publications on place identity from 1985 to July 2019. A total of 1,011 bibliographic records were obtained in the core data set of Web of Science with a title search for “place identity” or “regional identity” or “regional identities” between 1985 (the earliest year for data available in the core data set) and 2019.

There are many other scholars who have contributed a lot to enriching the meaning and theory of place identity (Relph, 1976; Tuan, 1977; Peterson, 1988; Saleh, 1998; Twigger-Ross et al., 2003; Carrus et al., 2005; Huigen and Meijering, 2005; Hauge, 2007; Groote and Haartsen, 2008; White et al., 2008). However, in most occasions, their conceptual foundation of place identity revolves on either the place identity of a place or people’s place identity. They seldom notice both sides of place identity, or at least, there is a paucity of empirical studies that have discerned the two sides of place identity. Academic journals have witnessed increasing publications in relation to place identity over the last 40 years, particularly since 2006, as shown in Figure 1. Nevertheless, few studies have reviewed and elaborated the versatile meanings of place identity. Limited arguments in a few studies are available on the relationship between these meanings. For example, Paasi (1986, 2002c) used “subjective identity of a region” to connect the two aspects of regional identity. The “subjective identity of a region” refers to images held by the people living in and outside the region, which resemble regional consciousness. Few studies proposed that individuals’ identification with a place can be reflected in the identities that they ascribe to the place and are subsequently incorporated into their own identities (Rijnks and Strijker, 2013). However, these intricate debates on the analytical interactions between place, people, and place identity make the meanings of place identity even more confusing. An additional obstacle to the understanding of place identity is the widely complained-about unclear relations between place identity and other environmental psychology concepts, such as place attachment, rootedness, sense of place, place dependence, and place satisfaction (Lewicka, 2011; Xu et al., 2015). Distinctions between these concepts have never been unanimously agreed upon and are still under discussion today.

Another point that might have also caused a fuzzy understanding of place identity is the interchangeable use of “region” and “place” in articles about place identity. Region is usually considered by geographers as a larger unit than place (Entrikin, 1991). Some scholars deem region as historically contingent social processes that occur at various spatial scales (Paasi, 1991, 1996, 2004). Compared with region, place is conceptualized more flexibly, without any presuppositions of scale. It has no essence but is open, fluid, and contested. Place is positioned in complex ways in power geometries and experienced in different ways by different people (Massey, 1994, 1995). The broad categories make “place” increasingly preferred by geographers today (Paasi, 2002b). While actors operate and (re)construct identities across scales, the link of identity to either the omni-scale region or the scale-free place can both hold and justify their positions (Paasi, 1996). Numerous articles titled with either “regional identity” or “place identity” can be searched in any journal database. Some of them may have a clear disciplinary background; for example, “regional identity” papers fit into geography, while “place identity” papers, into environmental psychology. However, there also exists chaos where mixed meanings of place identity are embodied in these two kinds of articles. Therefore, we include both of them in this review work, and in the rest of this article, the term “place identity” will be used primarily as a substitute for “regional identity.”

Different understandings of place identity and consequent divergent methods of measuring this construct make the accumulation of knowledge in this domain difficult (Lewicka, 2011). The emerging increment of publications in which numerous roles of place identity have been claimed makes it tough to grasp a coherent intellectual landscape of this area, if it exists. In order to synthesize the extensively studied place identities and their meanings, this work reviews how the literature has conceived and deconstructed place identity. We intend to guide audiences through the development of place identity since its introduction by Proshansky. The emphasis will be on sketching an overall intellectual landscape and investigating the active topics and new developments. More efforts will be paid to a deep examination of the measures and roles of place identity for tracing knowledge connections between different empirical understandings of place identity.

In the next sections, we first use CiteSpace to identify the active topics and new developments of publications on place identity. CiteSpace is a visual analytic system for visualizing emerging trends and critical changes in scientific literature (Chen et al., 2014a, b). Compared with a traditional literature survey, which relies on domain experts’ insights and selections of publications, CiteSpace adopts a computational and visual analytic approach drawn from the field of scientometrics and information visualization and can reach a much broader and more diverse range of relevant topics (Chen et al., 2014a, b). Our data set input into CiteSpace consists of 1,011 bibliographic records that are derived from the core database of Web of Science (WoS) with a title search for “place identity” or “regional identity” or “regional identities” between 1985 (the earliest year for data available in the core data base) and July 2019. Each bibliographic record contains the metadata of a published article, including the authors, the title, the abstract, the keywords, and the references with relevant information. In total, 31,307 references are cited by these articles. The examination of the measures and roles of place identity is based on some representative articles selected according to the analysis results generated by CiteSpace.

## Meanings of Place Identity

### People’s Place Identity

On an individual’s identity card, there is always information about where he or she is from. Everybody in the world belongs somewhere. Although the first impression an individual gives to others is always his or her physical appearance, such an impression would often coincide with the assumption of the place one comes from. For example, people with yellow skin and black hair would be judged as Asian. Therefore, we may realize that man is not simply a physical creature but mostly shaped by relationships, be they social, cultural, environmental, or others. The process by which other people judge you on your appearance is an expression of social and cultural relationships, as they are defining you as different from themselves. The influence that a place imposes on an individual’s identity is one of those relationships, and it constitutes part of the individual’s selfhood (Basso, 1996, p. 146).

In the late 1970s, to enlighten people on the importance of places influencing the formation of individuals’ identities, the term “place identity” was introduced by researchers within the interdisciplinary field of environmental psychology (Proshansky, 1976, 1978; Hauge, 2007). It is described as individuals’ incorporation of places into the larger concept of self (Proshansky et al., 1983). The work of Proshansky et al. (1983) has been criticized for their tendency to emphasize the individualistic dimensions of place identity (Dixon and Durrheim, 2000). This shortcoming has been mitigated by the later work on place identity, which suggested a genuinely social understanding of place identity by showing how places might become significant and contested arenas of collective being and belonging (Bonaiuto et al., 1996; Devine-Wright and Lyons, 1997; Dixon and Durrheim, 2000).

Despite the extensive studies on place identity after Proshansky, the theory of place identity is less developed (Bonaiuto et al., 1996). On the one hand, unlike gender, race, or nationality, which constitutes one category of identity, place contains symbols of many different social categories and personal meanings and represents and maintains identity on different levels and dimensions (Hauge, 2007). In other words, place is a component of diverse sub-identity categories, which makes the term “place identity” difficult to operate. When researchers make place identity the focus of their studies, they seldom follow the theoretical framework developed by Proshansky and his colleagues (Proshansky, 1978; Proshansky et al., 1983; Proshansky and Fabian, 1987). It is often referred as a term to describe a subjective feeling of identification with home, a neighborhood, or some larger space (Twigger-Ross et al., 2003; Carrus et al., 2005; White et al., 2008).

On the other hand, along with place identity, there are a number of other constructs developed by environmental psychologists to define and measure individuals’ relations with places, such as place attachment (Giuliani and Feldman, 1993), place dependence (Stokols and Shumaker, 1981), sense of place (Lalli, 1992), and so on. There has been no universal agreement on the relationships between these constructs (Fresque-Baxter and Armitage, 2012). White et al. (2008) saw place identity and place dependence as two dimensions of place attachment when they studied the effect of prior experience with setting and place attachment on visitors’ perceptions of recreation impacts. However, Bleam (2018) found that place identity is more than a sub-construct of place attachment. Hernández et al. (2007) viewed place identity and place attachment as different constructs. They thought place attachment develops before place identity. They contended that “one person could be attached to a place but not be identified with it,” and “someone could have a high personal identity with a place and not a high place attachment.” Hernández et al. (2010) argued that “place attachment is an affective–emotional bond with residence places, whereas place identity is a cognitive mechanism, a component of self-concept and/or of personal identity in relation to the place one belongs to.” Antonsich’s (2010) work pointed out the terminological confusion of these place-based concepts. He summarized that sometimes, place attachment is treated as a synonym of place identity, correlated with it or subsumed within it, or occasionally as a prerequisite. Sometimes, both place identity and place attachment are instead treated as components of sense of place (Jorgensen and Stedman, 2001). In other cases, place attachment is described as a superordinate category (Kyle et al., 2004). Chow and Healey’s (2008) argument is consistent with Antonsich’s findings. They noted that place attachment is subsumed under place identity in some cases, while some others view place identity as a form of attachment or consider both identity and attachment as dimensions of sense of place. This consensus is shared by Hernández et al. (2007) as well, who identified four different relations between place attachment and place identity according to their review of previous research. Other proposals suggest grading the sense of place. Shamai (1991) divided the sense of place into seven levels, in which attachment is a phase prior to identification.^1^

Confusion and overlap of the concepts that define emotional bonds to places, along with the multidimensional meanings of place itself, lead to the anxiety and criticism on the vague meanings of place identity (Hauge, 2007; Lewicka, 2008). Nevertheless, it has filled a gap in environmental theory and research, through bringing forth the idea that identity is formalized against the backdrop of physical environment (Bonaiuto et al., 1996; Hauge, 2007).

### Place Identity of a Place

Place identity ascribed by people to a place is constructed to differentiate one place from others. Differences between places are attributed or perceived by inhabitants living in or outside of those places. It is, to some extent, if not entirely, a subjective social construct based on objective physical settings.^2^ In this sense, the meaning of the construct “place identity” of a place resembles that of the construct individual’s “place identity” in that both of them are developed to describe subjective cognition about places.

As to the question of what place identity of a place is, the answer is still not clear, though it has been, for a long time, a frequently mentioned term in geographical research. Paasi (2001, 2002a,c, 2003, 2009b) argued that place identity refers to those elements of nature, culture, and regional life (inhabitants, people, or population) that distinguish a region from others. Groote and Haartsen (2008) defined place identity as a combination of physical and man-made processes, specific elements and structures in places, and meanings ascribed to places. The elements that have been referred to by studies as components of place identity indeed cover almost every aspect of a place. In other words, place identity can be anything that makes a place identifiable within the spatial system. There are no fixed components of place identity. Language is one of the most popular features to make places distinctive. Saleh (1998) used physical configuration of places or architectonics to describe place identity of Saudi cities, where local images are enhanced to meet individual and public needs. What’s more, people do not discern places in the same way. They perceive identities of places differently and differentiate them by drawing on different elements, such as physical features, cultural attributes, historical associations, experiential ties, and so forth (Peterson, 1988).

In many cases, the vague meaning and uncertain components of place identity make researchers map it by simply tracing unique features as indicators of place identity of that area. Paasi (2009b) criticized that it may lead to a rather “loose use” of place identity in research when any of these features is regarded as an illustration of place identity. People’s consciousness of a place should not be overlooked for understanding the identity of that place. Place identity comprises not only a material basis but also a “mental sphere” (Knapp, 2006). Place identity is formed after the place has achieved an established status in both the spatial structure of the society and its social consciousness (Paasi, 1991). From this view, Paasi (1986, 2002c) argued that subjective images and objective classifications are the two subcategories of place identity.

Compared with tracing indicators of place identity, the intention to claim place identity is sometimes considered as a more important matter (Billig, 1995). Identities are ascribed to a place by social actors who have different knowledge, interests, or power in that place. How place identities are constructed in discourses reveals partly the power balance between claimants in the political arena (Haartsen et al., 2000; Paasi, 2002c, 2003). Therefore, place identities are contested. What’s more, place identity should be understood as a dynamic process (Haartsen et al., 2000; Paasi, 2001). The formation of place identity is a process of shaping territorial boundaries, symbolism, and institutions (Paasi, 2003). Place consciousness can be strengthened during that process. Perceptions and understanding of the place identity of a place are often interpreted in narratives or discourses. Discourses about place identity are representations of claimants’ memories of the past, images of the present, and often, utopias of the future (Paasi, 2001).

## Active Topics and New Developments in Place Identity

### A Lightweight Survey of Major Topics

**FIGURE 2:**
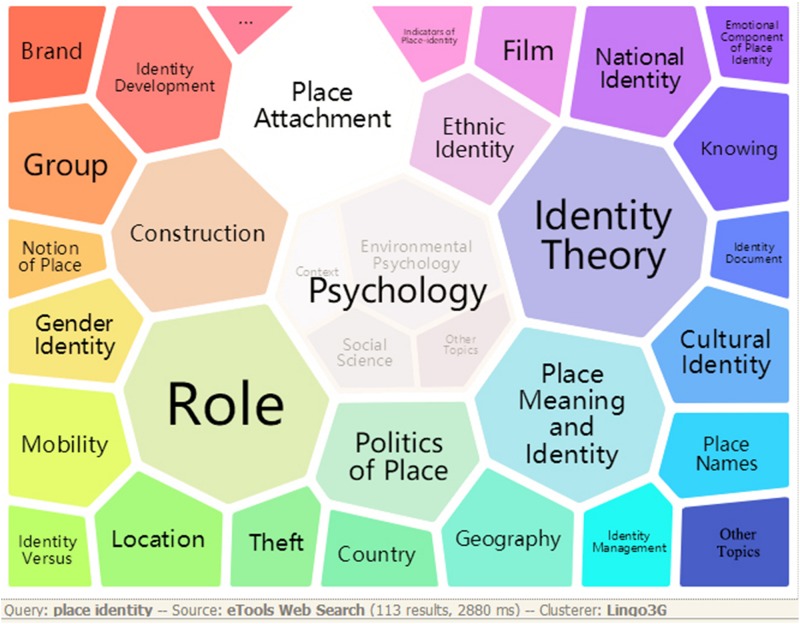
A foam tree map indicating the major topics in place identity on the Internet. The visualization was generated by Carrot2 workbench based on 113 search results of place identity.

**FIGURE 3:**
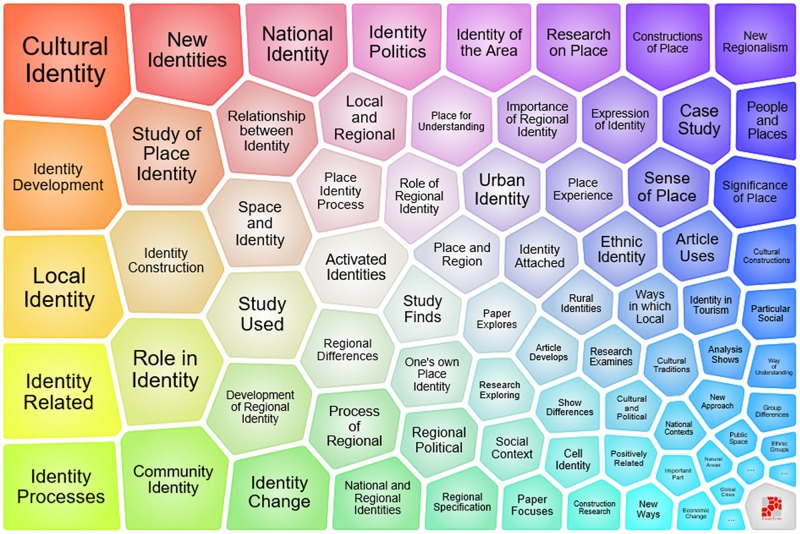
A foam tree map generated by Carrot2, indicating the major topics in place identity based on 1,011 bibliographic records about place identity in the core database of Web of Science.

Two polygonal tree maps (see Figures 2, 3), known as foam trees, were generated by Carrot2 to provide an instant and primary detection of major topics in the studies of place identity. Carrot2 is an open source search results clustering engine that can automatically organize small collections of documents from various search sources into thematic categories^3^. Figure 2 shows a foam tree based on the 113 results of a web search with the keywords “place identity.” The foam tree in Figure 3 was derived from the 1,011 bibliographic records of WoS, which were converted to the required format of Carrot2 prior to the data input.

With the views generated by Carrot2, the lightweight survey of major topics in the literature on place identity provides a useful basic reference to how the term “place identity” is generally conceived (Chen et al., 2014a). As shown in the two figures, *psychology, cultural identity, role, identity theory, identity development or processes, identity construction*, and *place attachment* are among the leading topics in the literature on place identity. Among other major topics, studies on place identity at different spatial scales constitute a certain portion of the data set, such as *community identity, urban identity, regional identity, national identity*, and *national and regional identities*. A distinction between the two aspects of place identity can be observed in the foam trees. Whereas topics such as *local identity*, *new identities*, *national identity*, *identity politics*, *identity of the area*, *importance of regional identity*, *role of regional identity*, *urban identity*, *development of regional identity*, *community identity*, *rural identities*, *place names*, and *regional specification* refer greatly to “place identity of a place,” topics such as *role in identity*, *identity processes*, *identity development*, and *one’s own place identity* have obvious connections to “people’s place identity.”

### Category and Keyword Co-occurrence Networks

**FIGURE 4:**
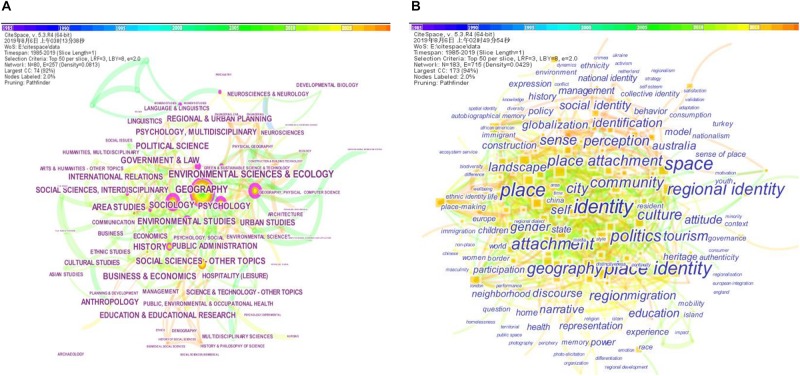
Co-occurring subject categories network **(A)** and co-occurring keywords network **(B)**. The color of the network edge indicates the year in which the co-citation link was first made. For example, early co-citations are colored in blue, and the latest are in yellow and orange.

Analyses of category or keyword co-occurrence networks can offer a more rigorous and reliable representation of the literature on major topics of a domain (Chen et al., 2014a, b; Shi and Liu, 2019). Each publication in the WoS is assigned one or more subject categories and a number of keywords. Through analyzing the input records with CiteSpace, a total of 81 unique subject categories were found (see Figure 4A). The size of a node in Figure 4A indicates the citation frequency of a category. For example, the largest node in Figure 4A is GEOGRAPHY, with the largest citation count value of 142, which means 142 publications of the data set fit in the category of geography. The top 10 subject categories ranked by citation counts are geography (142), environmental sciences and ecology (91), sociology (90), social sciences (87), psychology (82), environmental studies (77), history (71), area studies (67), government and law (66), and political science (64). In a visualization of CiteSpace, a purple ring is used to represent the degree of a node’s betweenness centrality. A node with high betweenness centrality indicates its important role of bridging the nodes it links. The top 10 subject categories ranked by betweenness centrality are psychology (0.30), sociology (0.28), environmental sciences and ecology (0.27), psychology—multidisciplinary (0.22), environmental studies (0.19), psychiatry (0.18), geography (0.16), genetics and heredity (0.14), education and educational research (0.13), and regional and urban planning (0.12). A total of 183 unique co-occurring keywords were found (see Figure 4B). The top 10 keywords ranked by citation counts are identity (109), place (82), place identity (71), space (68), regional identity (43), politics (41), attachment (41), geography (35), community (32), and place attachment (30). From the results in Figure 4, we can observe a general knowledge base that supports the research of place identity. As an interdisciplinary subject, place identity obtains its primary foundation in geographical sciences. Under the bridging efforts of psychology, sociology, and environmental sciences, it develops in other relevant domains, for example, government, political science, or spatial policy. It is worth noting that place attachment and place identity co-occur frequently as keywords, which indicates that there are considerable debates in the literature on their close and obscure relationship.

**FIGURE 5:**
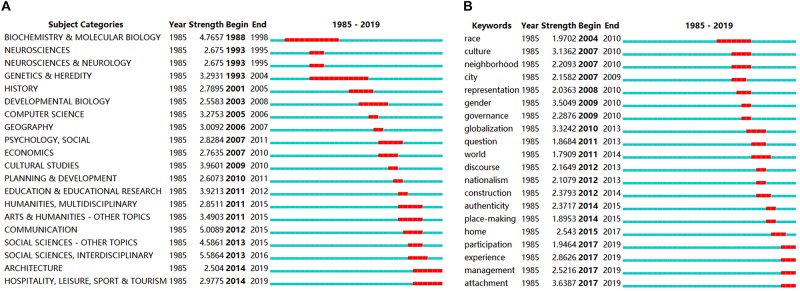
Top 20 subject categories **(A)** and keywords **(B)** with the strongest bursts.

As shown in Figure 4, the research on place identity involves numerous disciplinary areas, which may shift over time. The burstness, signifying the fast shift of subject categories and keywords, indicates the most active research topics (Chen et al., 2014a, b). Figure 5 shows the top 20 subject categories and keywords with the strongest citation bursts. The refined review results of the subject categories in Figure 5 reveal the neurobiological supports for the phenomenological studies of human geography and environmental psychology on place identity (Lengen and Kistemann, 2012). At the top of the list of Figure 5A, the discipline of biology, including the subject categories of *biochemistry and molecular biology, neurosciences, neurosciences and neurology*, and *genetics and heredity*, occupied the hot area of place identity study prior to 2000. The decade after 2000 experienced an active exploration in plural disciplines in research on place identity, such as *history, computer science, geography, psychology, economics, cultural studies, planning*, and *education*. Gradually, the hot area came to the *humanities* and *social sciences* after 2010, while the subject category *social sciences—interdisciplinary* obtained the strongest burst strength of 5.5864 in 2013–2016. Recent hot areas were captured by *architecture*, *hospitality*, *leisure*, *sport*, and *tourism*, which suggests a shift of the study focus of place identity toward place products or place marketing. In general, the development of place identity research mainly experienced four stages, namely, theory construction and verification, active multidisciplinary exploration, in-depth study in social sciences, and application of place identity theory in place development.

Bursts of keywords may provide a more refined indication of hot topics (see Figure 5B). All of the top 20 keywords bursts appeared in the last 15 years. The keyword *attachment* has a strong burst from 2017 to 2019, with the maximum burst strength 3.6387, and it is used in 41 articles. It seems the keywords on the list can be classified into four groups. The first group focuses on places of various scales, including *home*, *neighborhood*, *city*, *nationalism*, and *world*. The second group focuses on personal characteristics, attitudes, and behaviors, including *race*, *gender*, *participation*, *experience*, and *attachment*. The third group focuses on the construction of place identity, including *construction*, *place-making*, *representation*, and *discourse*. The fourth group focuses on globalization and countermeasures, including *culture*, *authenticity, globalization*, *governance*, and *management*. The above preliminary classification implies that the research problems of place identity mainly revolve around *where* the place identity represents, *whose* place identity it is, *what* the place identity is, and *how* the place identity is affected.

### Clusters of Co-cited References

**FIGURE 6:**
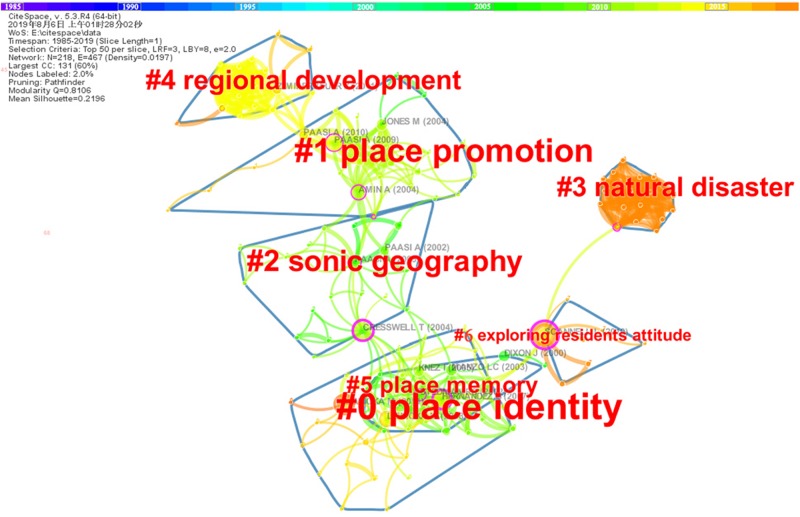
A network of co-cited references based on citation instances made by the top 50 most-cited articles per year between 1985 and 2019.

**FIGURE 7:**
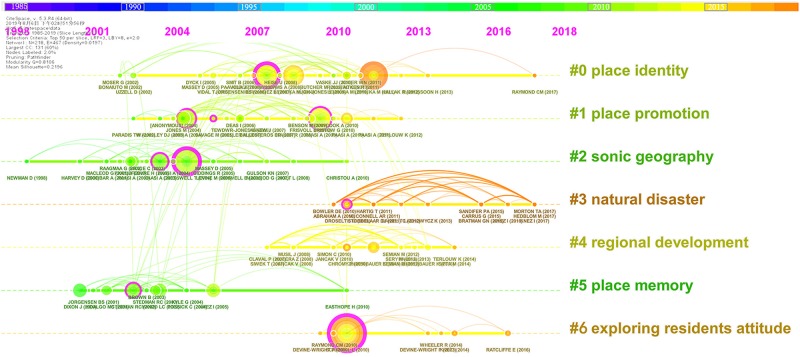
A timeline visualization for the clusters between 1985 and 2019. New developments since 2010 are notably associated with cluster #3, #6, and #4.

CiteSpace provides visualizations of synthesized networks of co-cited references, which are usually used for the analysis of emerging trends and new developments of a research field (Chen et al., 2014a, b). Figure 6 shows a snapshot of the network of co-cited references derived from the core data set with CiteSpace. A link in the network represents how frequently two articles are cited together by other articles in the data set. A red ring in the network denotes a citation burst, while a purple ring represents the betweenness centrality. The size of a node of the network indicates the frequency of citations of an article. Based on their interconnectivity, these nodes can be aggregated into clusters that represent thematic concentrations. A cluster is a group of tightly coupled references that form the intellectual base of a research field, and articles citing these references represent the research front of that field (Chen et al., 2014a, b; Shi and Liu, 2019). The seven largest clusters numbered from 0 to 6 are shown in Figure 6. Table 1 summarizes the basic information of the seven clusters. The label of each cluster is extracted with a log-likelihood ratio (LLR) test, and it summarizes the impact of the cluster on more recent research (Chen et al., 2014b). A silhouette value ranging from the lowest, -1, to the highest, 1, is used to measure the homogeneity of a cluster. Each of the silhouette scores in Table 1 is close to 1, suggesting a reliable quality of grouping. Timeline visualizations generated by CiteSpace can make the new developments in research on place identity more easily recognized. As shown in Figure 7, the most recently emerging area is captured by cluster #3, labeled *natural disaster*, which can also be confirmed by the average year in Table 1, which indicates the average publishing year of the cited references of a cluster. Cluster #6 on *exploring residents’ attitude* and cluster #4 on *regional development* are also relatively new.

**TABLE 1:** Largest clusters of co-cited references.

**Cluster ID**	**Size**	**Silhouette**	**Average year**	**Label (log-likelihood ratio, *p*-value)**
0	31	0.881	2008	Place identity (788.36, 1.0 × 10^–4^)
1	25	0.925	2007	Place promotion (473.96, 1.0 × 10^–4^)
2	21	0.884	2003	Sonic geography (483.58, 1.0 × 10^–4^)
3	17	0.994	2013	Natural disaster (397.53, 1.0 × 10^–4^)
4	17	0.963	2010	Regional development (609.67, 1.0 × 10^–4^)
5	13	0.952	2003	Place memory (352.53, 1.0 × 10^–4^)
6	7	0.988	2012	Exploring residents’ attitude (125.06, 1.0 × 10^–4^)

Among these seven clusters, the largest one is cluster #0, labeled *place identity*, which contains 31 member references. Most of the top cited references in cluster #0 discussed place attachment, and some of them debated its relationship with place identity (Hernández et al., 2007; Lewicka, 2008, 2010, 2011; Rollero and Piccoli, 2010). Articles citing members of a cluster determine the aggregations of co-cited references, and they indicate major interests and research trends in a thematic field associated with the cluster (Chen et al., 2014a, b). Table 2 shows the most-representative citing articles of the seven clusters. CiteSpace identified 11 citing articles shaping cluster #0. Evident thematic connections revolving around place identity can be observed from the titles of these citing articles. Particularly, these connections reveal the research concentration of cluster #0 on the role of place identity in social actors’ attitudes and behaviors in the present transitional world with environmental depredation, climate change, urban renewal, and increasing and complex human needs, if we look further into the contents of these articles.

**TABLE 2:** Articles that cite over 10% of members of each cluster.

**Cluster ID**	**Coverage %**	**Citing articles**
0	19	Fresque-Baxter and Armitage, 2012
0	19	Hernández et al., 2010
0	13	Vidal et al., 2013
1	16	Zimmerbauer, 2011
1	12	Vainikka, 2012
1	12	Zimmerbauer et al., 2012
2	24	Boland, 2010
2	19	Simon et al., 2010
3	24	Knez et al., 2018a
3	24	Knez et al., 2018b
4	53	Semian and Chromy, 2014
5	38	Lewicka, 2008
5	15	Chow and Healey, 2008
6	29	Bradley, 2017
6	29	Wheeler, 2015

The second largest cluster, #1, shows thematic focus on the construction of place identity in the process of place promotion. The term “place promotion” appears in the title of a top citing article (Zimmerbauer, 2011). Place promotion and marketing are keystone practices in regional development, where political forces always play a determinant role. Deinstitutionalization or institutionalization of a region imposed by politics may lead to regional exclusion or othering when place promotion strategies are taken. Spatial identities are constructed by various stakeholders at different spatial scales. Place identity emanating from residents’ perspectives has attracted a lot of attention in this theme, whereas its construction is still essentially impacted by the globalizing forces of development.

For cluster #2, the term “sonic geography” in the title of the top citing article (Boland, 2010) is extracted to be the group title. In the article, the influence of sound, for example, a distinctive accent and/or dialect, on the construction of local identity is examined. The cited references of cluster #2 concentrate highly on Paasi’s articles (Paasi, 2002b, c, 2003), which results in the thematic focus of this cluster on the construction of place identity of regions. Research emphases of cluster #2 have been put on special factors shaping place identity, for instance, vernacular, symbols, narratives, children’s reading and writing, and professionals involved in the development of a region.

The young cluster #3 focuses on the effects of physical environment (change) on place identity and well-being. Knez et al. (2018a) article titled “Before and After a Natural Disaster: Disruption in Emotion Component of Place-Identity and Wellbeing” gained much attention in this cluster. Studies on the role of place identity in regional development are prominent in cluster #4. Place identity may play a driver or a barrier to regional development. Graphic symbols, landscape features, discourses, rurality, and other place components can be employed as identity instruments for place marketing and local development. Yet, the mechanism underlying the role of place identity construction or reconstruction in regional development is under-discussed and needs more study efforts.

Representative citing articles of cluster #5 suggest a thematic concentration on the effects of socio-spatial environment (change) on place identity. The term “place memory” for the cluster label appears in the title of a top citing article (Lewicka, 2008). The shaping of memory depends on many socio-spatial environment factors, such as history, culture, architecture, and family, which overlap the components shaping place identity. The transition or new development of a socio-spatial environment, for instance, undergraduates making the transition from home to university, can disrupt pre-existing memory and emotional attachments to a place and threaten place identity processes. The smallest cluster, #6, includes seven member references. Cluster #6 pays special attention to the relationship between place identity and residents’ attitudes, especially the attitudes toward spatial planning or human intervention in places.

Apart from the difference in thematic concentrations, these seven clusters also differ in the foundation of the meaning of place identity upon which the citing articles are based. In Table 2, representative citing articles of cluster #0, cluster #3, and cluster #5 conceive place identity as an individual’s sub-identity. Those of cluster #1, cluster #2, and cluster #4 generally deem place identity as a social construct that differentiates a place from other places. However, place identity in the article by Zimmerbauer (2011) in cluster #1 is understood more in terms of regional consciousness and a sense of belonging to a region; a similar understanding can be found in the citing article by Miloslav (2018) in cluster #4. Some citing articles, for instance, Antonsich (2010) in cluster #2 and Semian and Chromy (2014) and Melnychuk and Gnatiuk (2018) in cluster #4, advocate the co-existence of the two intertwined and complementary meanings of place identity. The two representative citing articles of cluster #6 do not share the same view on the meaning of place identity. Wheeler (2015) adopted a similar meaning as that of cluster #1, cluster #2, or cluster #4. In the article by Bradley (2017), the meaning of place identity as a human’s sub-identity seems mixed with that as features of a place. Though versatile meanings of place identity are used in different clusters or even in articles of a single cluster, they are not isolated and hence are sometimes intertwined in shaping the knowledge base of thematic concentrations. For example, Stedman’s (2002) article titled “Toward a Social Psychology of Place: Predicting Behavior From Place-Based Cognitions, Attitude, and Identity,” which has a betweenness centrality score of 0.11 and the most-cited frequency in cluster #5, is also co-cited in cluster #0 and cluster #2.

**FIGURE 8:**
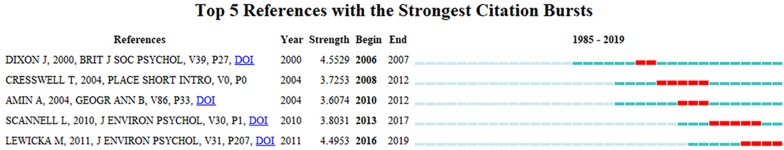
Top five references with the strongest citation bursts during 1985–2019. The red line segment indicates the beginning year and the ending year of the duration of the burst.

The extensive applications of place identity in various topics and the complex connections between its meanings in articles or thematic clusters have strengthened the necessity of in-depth examination on its fundamental meanings. Indeed, such necessity has already been responded to in the academic world, which is revealed by our citation burst analysis. A citation burst is a phenomenon where an article is highly cited at an increasingly faster rate. It indicates the likelihood that the scientific community has paid or is paying special attention toward the underlying contribution of these cited articles (Chen et al., 2014a, b). The top five cited references with the strongest citation bursts associated with Figure 7 are listed in Figure 8, out of which Dixon and Durrheim (2000) is in cluster #5, Lewicka (2011) in cluster #0, Scannell and Gifford (2010) in cluster #6, Cresswell (2004) in cluster #2, and Amin (2004) in cluster #1. Dixon and Durrheim’s (2000) article titled “Displacing Place-Identity: A Discursive Approach to Locating Self and Other,” has the strongest citation burst, with a burst strength of 4.55. This article, published in *British Journal of Social Psychology*, presented a sympathetic but critical evaluation (review) of research on place identity. The beginning year of the citation burst of this article is 2006, which coincides with the time when the acceleration of publications on place identity took off (see Figure 1). The articles by Amin (2004) and by Cresswell (2004), both of which elaborated the concept of place, attracted great attention in studies on place identity from 2008 to 2012. The latest citation bursts are the remaining two review articles published in the *Journal of Environmental Psychology* by Scannell and Gifford (2010) and Lewicka (2011) on place attachment, in which its relation with place identity is discussed. From the evolvement of these top five busts, we can see that the fundamental meanings of place identity have been widely of concern in studies in this field. Articles focusing on reviewing or elucidating the meanings of place, place identity, and other place-related concepts have gotten critical attention. The attempts to fit together the versatile meanings of place identity have witnessed increasing difficulties, as the meanings of place, other place-based concepts, and their confusing interrelations have been developing.

## Measuring Methods and Roles of Place Identity

Our scientometric survey has revealed the intellectual structure of the research landscape relevant to place identity. However, there are still some key issues about place identity research that this methodology seems unable to answer. For example, how has place identity been measured in the literature? What are the roles of place identity that have been identified? The method to measure or reveal place identity can be most scholars’ primary concern when they propose questions in this domain. The roles of place identity in relation either to people or to regions have long been debated (see cluster #0, cluster #4), yet no universal agreement has been reached. In this section, we reviewed these two essential issues by further examining the contents of articles on place identity.

### Measuring Place Identity

#### People’s Place Identity

As a subjective and enigmatic social construct, place identity is seldom engaged in individuals’ daily thoughts and communications. People do not become aware of place identity until their sense of place is threatened (Proshansky et al., 1983). Individuals’ lack of self-awareness of place identity has not discouraged researches on place identity assessment. It is shown in plenty of articles that place identity can be measured either qualitatively or quantitatively.

As witnessed in the citing articles of cluster #3 and cluster #5, changes or interventions in living environment usually offer good opportunities for researchers to investigate the affected individuals’ place identity. Chow and Healey (2008) interviewed 10 students twice over a 5-month period to assess the extent of changes in their place identities. These students had relocated from their homes to university. After the hurricane Hugo in September 1989, Hull et al. (1994) interviewed the residents of Charleston by telephone about the meanings associated with physical features damaged or lost due to Hugo. The authors argued that the meaning and values symbolized by place features or place icons play a role in the formation of place identity.

Rank ordering of place-related self-categorizations is widely used to assess place identity in many countries. The top citing article of cluster #5 (Lewicka, 2008) applied this instrument in a study on memory of residence places and its relationship with place identity and place attachment. Participants in the study were asked to rank three objects out of a list of possible places of identification, which include city district, city, region, country, Europe, the world, and finally, a human being.

A lot of studies rely on a Likert scale to assess individuals’ place identity (Kyle et al., 2004; Williams and Roggenbuck, 1989; White et al., 2008). In many cases, these Likert-scaled items are measured on a five-point strongly agree to strongly disagree response scale. Typical items include “the place means a lot to me,” “I am very attached to the place,” “I identify strongly with this place,” “I have a special connection to the place and the people who live and visit there,” and so forth. In some studies, such items are measured with a seven-point scale. Carrus et al. (2005) measured regional identity with 16 items on a seven-point Likert scale. The authors divided the items into two categories, regional pride and regional empowerment. In a citing article in cluster #3 by Knez et al. (2018a, b), the 10 statements to measure place identity are also divided into two categories, namely, emotion and cognition components, and responded to by participants on a seven-point scale.

Some studies have made attempts to measure place identity with a single question. Pretty et al. (2003) asked for residents’ responses to a statement to test their identification with their residential community. The statement was designed as, “I would really rather live in a different town. This one is not the place for me” (p. 275). The authors argued that “one’s town is not the place ‘for me’ is to suggest that one’s town is not constituted as part of one’s self-identity” (p. 275). Respondents who agreed with the statement were classified into a group with low community identity. Those who disagreed were classified into a group with high community identity. The community identity of undecided respondents was deemed undecided.

#### Place Identity of a Place

Scholars tend to use “strong” or “weak” to depict the intensiveness of people’s perception of the place identity of a certain area. In this sense, there can be a scale to measure the intensity of place identity. However, such a scale has not yet been unanimously agreed on in practice. Various methods have been adopted in literature to trace identities of places.

Approaches in literature to depict and measure place identity focus generally on what the identity is and how it is constructed by different social actors. Vainikka (2012), a representative citing article in cluster #1, used focus-group interviews to scrutinize to what extent place identities are shaped and shared by citizens. The author found that place identities perceived by citizens often differ from the identity discourses produced by media, regional administrations, and others. Haartsen et al. (2003) proposed a method to assess the rural identity perceived by residents of different age groups in the Netherlands. They asked the respondents to give the four words or phrases that first come to their minds when thinking about the countryside. The answers were aggregated into three categories: (1) a socio-economic functional image base (“how rural areas work”), (2) a visual–figurative image base (“what rural areas look like”), and (3) a socio-cultural image base (“what rural areas mean”). Elderly people were inclined to have a more socio-cultural representation of rurality, whereas younger people often characterized the countryside as an agricultural production zone. Similarly, place identity is divided into three interpretative dimensions, strategic, cultural, and functional, in the article by Van Houtum and Lagendijk (2001). This division was applied as a place identity analysis framework in two regions with comparable economic conditions and urban structures. The authors captioned each identity dimension of the two regions and elaborated with facts and historical narratives on how that identity was constructed.

As discussed previously about the thematic focus of cluster #2, extracting place identity components from texts, discourses, and narratives about an area is widely used by studies in this domain. Ritalahti (2008) searched and analyzed the web pages of individual tourism enterprises, municipalities, and other actors, in order to get a holistic view of how the identity of the study area is described there. The author did the same analysis in neighboring areas to detect possible boundaries created with place identities by social actors between these areas. Visitors’ perceptions of the study area were then compared with the place identities ascribed by the tourism actors. Brace (1999) examined the identities of Cotswolds and the larger England in fictional and non-fictional rural writing, topographical writing, guidebooks, magazines, and articles. Other publicly available materials in relation to place identity, such as advertisements, were analyzed by researchers as well. Keegstra (2009) collected the pictures in advertisements on websites from different social actors in the Loire valley in France. The author identified different identities ascribed by different groups, including tourist organizations, farmer organizations, and governments.

Some research uses photos to measure how people perceive a place. This instrument is generally applied in two kinds of approaches. In the first approach, researchers take photos and present them to respondents (Van den Berg, 1999). Buijs et al. (2009) asked respondents to assess full-color pictures of 10 typical and non-urban Dutch landscapes to analyze the cultural differences in landscape perceptions in the Netherlands. Each picture was scored on a 10-point scale, ranging from 1 (not attractive at all) to 10 (extremely attractive). Hawthorne et al. (2008) applied a similar approach to examine residents’ perceptions of land use in their neighborhood. The participants were asked to rank 19 pictures related to trail development and explain why they ranked them in that way, which was intended to understand the image of the neighborhood in residents’ mind. In the second approach, researchers asked participants to take photos that they think can represent the identities of a place. Stewart et al. (2004) used this “photo-elicitation” method to map residents’ visions of their community. Twenty participants took photographs of community landscapes and were interviewed while viewing their photographs. The interview texts about meanings of the environments were analyzed and grouped.

In addition to texts (discourse or narrative) and photos, other identity indicators, known as identity markers, are also explored extensively to reflect the distinctive identities of a place. Such identity markers include buildings, street symbols, landscape, cultural traditions, region names, dialects, dressing, and so forth, most of which can be found in the citing and cited articles of cluster #2, cluster #4, and cluster #6. Saleh (1998) argued that citizens recognize their places, to some extent, by the prominent traditional structures in the built environment. They examined the role of the various kinds of towers in constructing the identity of Saudi. Peterson (1988) noted that “people perceive places differently and differentiate between them by drawing on key physical features, cultural attributes, historical associations, experiential ties” (p. 451). He explored the place identity of Tucson and Albuquerque by analyzing thousands of commercial and non-commercial establishment names listed in the telephone directory. Nogué and Vicente (2004) suggested that landscape is the cultural projection of a society on a place and acts as a center of meaning and symbolism, and is a fundamental element in the constructing process of a territorial identity. They analyzed the role of landscape in the creation of national identity in Catalonia. Simon et al. (2010) argued that names of regions are essential symbols of place identities, and they connect the image of regions with people’s regional consciousness. Social actors may try to improve the identity of a place by giving it a new name that can be associated with a positive image of the place. The authors traced and counted the names of regions in the Netherlands in the period 1950–2000 for an analysis of change in Dutch place identity. Wheeler (2015) investigated how wind farms are perceived by local residents as a reference to rural place identity.

### Roles of Place Identity

#### People’s Place Identity

People’s place identity is individuals’ strong emotional bonds to particular places or environments (Fresque-Baxter and Armitage, 2012; Zimmerbauer et al., 2012; Melnychuk and Gnatiuk, 2018). It influences the way we look, see, think, and feel in our interaction with the physical world. Proshansky et al. (1983) has been frequently cited in the clusters that concentrate on people’s place identity, such as cluster #0, cluster #3, and cluster #5 (Chow and Healey, 2008; White et al., 2008; Devine-Wright, 2009; Devine-Wright and Clayton, 2010; Hernández et al., 2010; Fresque-Baxter and Armitage, 2012; Lengen and Kistemann, 2012; Urquhart and Acott, 2013). In this article, the authors initially depicted that place identity has five core functions: *recognition*, *meaning*, *expressive-requirement*, *mediating change*, and *anxiety and defense* function. The *recognition* function implies that individuals may adapt to and derive satisfaction from the settings where they spend a while. The *meaning* function proposes that place identity is the source of meaning for a given setting because of the cognitions that enable the person recognize a setting and understand its purposes. The *expressive-requirement* function involves two types of place identity cognitions, of which one is a cluster of cognitions that expresses the tastes and preferences of the person and the other represents what spaces and places require as far their purposes are concerned. The *mediating change* function indicates those place identity cognitions that serve to mediate change in discrepancies between a person’s place identity and the characteristics of an immediate physical setting. The *anxiety and defense* function refers to those place identity cognitions that may signal threat or danger in physical settings or may represent response tendencies that defend or protect the person against these dangers.

There are many other efforts to explore the roles of place identity. For example, Fresque-Baxter and Armitage (2012), a representative citing article of cluster #0, summarized three conceptual approaches to analyze the role of place identity in climate change adaption. The first approach is the cognitive–behavioral approach, which examines how place identity influences individual decision making from risk perception to intention to action. The second one is the health and well-being approach, which emphasizes the importance of place identity in individuals’ emotional, mental, and spiritual well-being. The third one is the collective action approach, which examines the role of place identity in shaping values at a collective level and opportunities for and/or barriers to collective action. However, within the literature, the functions of place identity articulated by Proshansky et al. (1983) or others are seldom systematically testified or refined in empirical studies. In some occasions, part of these functions may match or relate to the assumptions or conclusions of a few studies, either because these functions are defined inclusively or because they fit well with the real world.

Among empirical studies on place identity, most of them have demonstrated its positive effects, which include preventing negative environmental perceptions, providing support for public land use policies, strengthening the commitment of residents to improve their homes and neighborhoods, and so forth. By examining the effects of nationalism and local identity upon perception of beach pollution, Bonaiuto et al. (1996) found that identification with a town or nation prevents people’s negative physical assessments of pollution, which is interpreted as a strategy used to cope with the threat by an outgroup to place identity. Low and Altman (1992) identified “satisfaction” as part of the merits of people’s affective bonds with places because “places permit control, foster creativity and provide opportunities for privacy, security and serenity” (Chow and Healey, 2008). Kyle et al. (2003) found that when visitors’ place identity increases, their support for paying fees for recreational use increases as well. Uzzell et al. (2002) argued that place identity can positively predict environmentally friendly conduct. Brown et al. (2003) found that the commitment of neighbors to improve their own home and the whole neighborhood is positively related to the emotional connection they have developed to the place. Carrus et al. (2005) also detected a positive role of place identity in predicting support for the protection of nature areas. Manzo and Perkins (2006) argued that particular preferences, perceptions, and emotional connections to places relate to community social cohesion, organized participation, and community development. Hernández et al. (2010) found that place identity positively influences environmental attitude and injunctive social norms. Knez et al. (2018b) found a positive relationship between place identity and perception of naturalness.

A few articles have also pointed out that the strong emotional bonds between individuals and particular places may have a less positive role. As place identity is a dynamic and dialectic process, in some occasions, it may act as resistance factors that are latent in man’s mind. Manzo and Perkins (2006) argued that if people’s emotional responses to a place are not acknowledged and understood, they can be divided and immobilized by their anxieties. Forester (1987) pointed out that bonds with a place can form the basis for cooperation and community action, and they can also lie at the root of community conflict. Both Stoll-Kleemann (2001) and Bonaiuto et al. (2002) suggested that local residents would oppose the designation of protected nature areas if a strong sense of identification with their local community is present.

#### Place Identity of a Place

It is widely accepted and also revealed in cluster #4 and cluster #1 that place identity is closely related with regional development. Paasi (2009b) argued that a strong place identity is a remarkable resource in regional development. Place identity is the image inhabitants hold of the home area, and the embodiment of such an image in regional development activities encourages people’s creativity and entrepreneurship. Semian and Chromy (2014), the top citing article of cluster #4, shared Paasi’s conceptualization of place identity and argued that place identity can play a positive or negative role in regional development. Terluin (2003) did not mention place identity as a determinant of leading and lagging EU regions, but she believed that strong and leading actors’ capacity is vital for regional development in building a strong place identity, which can facilitate the mobilizing of the self-help capacity, foster co-operation, and help turn handicaps into development assets. Taking EU as an illustrative case, Paasi (2009b) pointed out that the emotional aspects of civil society are increasingly recognized in the cohesion policy of the EU, and place identity has become an instrument for promoting regional development. The role of place identity as a tool to promote regional cooperation and possibly regional integration has been witnessed in East Asian as well (Terada, 2003). However, place identity is broadly considered as an inherently imprecise and fuzzy notion, and the mechanism about how place identity affects regional development is laden with social and productive magic (Hurrell, 1995; Paasi, 2003). Though social actors’ commitment to enhancing place identity, and subsequently, regional development, is widely acknowledged, the principles behind such contribution have seldom been proven (Terluin, 2003). There is a long way ahead for scholars to identify how and to what extent place identity can promote regional growth.

Numerous studies on place identity have stressed its intrinsic nature of resistance to globalization in the process of regional development, which responds to our previous keyword burst analysis, where globalization and countermeasures are highlighted as hot topics (Paasi, 2002c, 2003; Van Rekom and Go, 2006). Globalization can be defined as the intensification of economic, political, social, and cultural relations across borders, while regionalization is the growth of societal integration within the border of a given region (Kacowicz, 1999). A region is established in the regionalization process, maturing with people’s consciousness of place identity that differentiates it from others. While increasingly fast globalization is threatening the genuineness of regions, place identities stand to maintain regional distinctiveness. This plausible contention can be greatly found in research about tourism and place identity. Dredge and Jenkins (2003) argued that globalizing forces have contributed to increased homogenization of tourism products. Local destination identity has been associated with perceptions of homogeneity. Destinations are under increasing pressure to construct and promote distinct identities in order to counterbalance these homogenizing influences and pursue tourism growth. Gospodini (2004) suggested that built heritage and innovative design may work as place identity generators in modern and post-modern European urban societies. They can promote tourism and create social solidarity among inhabitants, and subsequently defend against the place identity crisis in the processes of economic and cultural globalization. While the preservation of place identity can attract tourists, it puts the place at a risk of exposure to globalization forces. However, economic success brought by tourism may stimulate the place to maintain its identities that are discovered by tourists (Van Rekom and Go, 2006).

Besides the role in maintaining a place, place identity has also become a slogan for making a place, which is also referred to in our previous keyword burst analysis (Paasi, 2009b). For one, an established place identity shared by common social stakeholders forms structures of expectations, which can be used as a collective mobilizing force (Terluin, 2003; Knapp, 2006). For another, discourses on place identity, which embody social actors’ interests and expectations, may create the reality that they are describing and generate action when the reality is accepted by the public (Paasi, 2002a). Politicians and policy makers have been, for a long time, exploiting place identity narratives in planning to lead place-making (Paasi, 2009b). Saleh (1998) argued that place identity is an important factor to mobilize people, which explains planners’ enthusiasm for its introduction and application. Stewart et al. (2004) suggested that place identity has the potential to serve as visions for planning processes. The involvement of citizens’ thoughts on what the identity of a place is and should be forms part of the visioning process in planning. Hague and Jenkins (2005, p. 8) deemed planning to be about place-making, which means one of the significant objects of planning is to “create, reproduce or mold the identities of places through manipulation of the activities, feelings, meanings and fabric that combine into place identity,” Moreover, place-making needs planners to incorporate visions of place identity not only from politicians’ and economic interests but also from local residents and other members of civil society, because identities ascribed to a place by different stakeholders are contested and may play a potential role in social conflicts (Bridger, 1996; Haartsen et al., 2000; Stewart et al., 2004).

## Discussion

Place identity is gradually preferred by geographers to preach in extensive occasions, such as planning, regional regeneration, heritage conservation, landscape appreciation, tourism, environmental management, environmental behavior, local conflict, and so on. Researchers involve place identity in so many diverse contexts that this term is endowed with increasing explanations and connotations. The broad use of place identity makes it like a panacea to deal with problems of the relationship between a place and people. Although the meanings of place identity have not been unanimously agreed upon to date, it is undoubtedly positive that “place identity” has acted as an outlet to integrate physical reality and social cognition. When we begin to become aware of the complexity and chaos in place management issues, the object-oriented strategy-making process fades out of its dominant popularity in place-making. As a traditional top–down policy-making approach tends to meet resistance in the late policy implementation phase, increasing attention has been paid to information and knowledge from the grassroots and stakeholders. Participatory planning has been introduced, and a great deal of consultancy work is launched before decision making. To do that, we aim at digging about what and how people think of the place where they live or that they care about. People’s perception of a place derives from direct or indirect contact with the place. They ascribe identities to a place based not only on objective physical features but also on less tangible meanings, memories, and information from others, from the past and the future.

One of the essential prerequisites to make a place is to cater to its owners’ or users’ thoughts and requests. The rising of inhabitants’ versatile requests on places drives us to contemplate place management issues as an integration of complexity. We want to modernize our place with global leading technologies, yet we are afraid of losing its historical traces. We need wide roads to improve traffic and larger cities to accommodate more population and buildings, yet we are reluctant to sacrifice limited farmland and natural landscape, which preserve rural identity. Such dilemmas or conflicts in place development are realities, and we cannot tackle them within a single sector or discipline. Place identity can be a solution to integrate those issues (Pinto, 2000). However, when we come to this approach, we may encounter another risk. Since the components of place identity are too broad and inclusive to operate, pursuers of place identity are sometimes frustrated by its vague meaning. In a lot of studies, researchers try to avoid in-depth debate on the conception of place identity when they investigate identities of a case area. Instead, they lean on previous relevant studies and extend those definitions and methodologies in their own ways.

**FIGURE 9:**
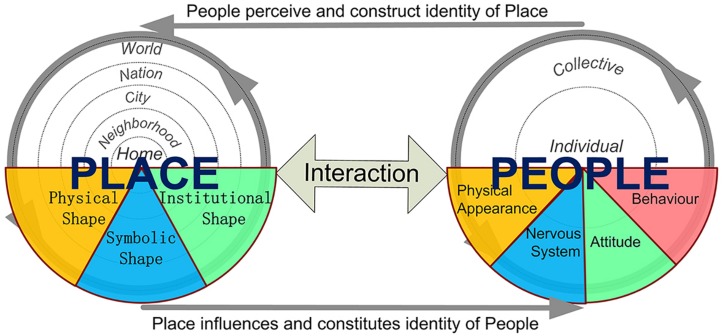
Relationships between people, place, and place identity.

People’s place identity and the place identity of a place overlap but are not the same. Both constructs embody subjective or emotional bonds between man and the physical world. People’s place identity is part of individuals’ personalities related to places that are significant in the formation of their identities. Place identity of a place is the personality of the place. Such personality is, in most occasions, ascribed by people to the place where they live or that they care about. As shown in Figure 9, through people’s interaction with a place, the place influences and subsequently constitutes people’s social (collective) and personal (individual) identity (Nario-Redmond et al., 2004). Meanwhile, people perceive and construct the identity of a place. Although the identity of a place is reflected through people’s consciousness, which is generated mostly by man’s nervous system, it originates from the physical, symbolic, institutional, and other components of the place (Raagmaa, 2002). Any changes in these components, deduced either by external forces (e.g., natural disaster, spatial planning, globalization) or internal growth (e.g., regional development or promotion) would impact the identity of a place and its inhabitants’ place identities. As the interaction between people and a place is a mutual, dynamic, and eternal process, the creating and fostering of place identity is also a mutual, dynamic, and circular process (see Figure 9) (Ramos et al., 2016).

Generally, the efforts to promote the identity of a place can enhance its inhabitants’ place identities at the same time, and vice versa. The intricate relationships between people, place, and place identity occasionally confuse researchers who try to deconstruct the meanings of place identity. For example, Yuen (2005) cited Proshanky’s definition to conceive place identity as a “substructure of self-identity.” However, most of the arguments in his article concentrate on the increasing emphasis that Singapore has given to urban conservation as a way to strengthen the identity of the city. Paasi’s theory on the formation of a region and its identity is applied in the research by Raagmaa (2002). The measurement of place identity Raagmaa designed is biased to examine residents’ identification with their communities. We need place identity as a term to integrate complex physical reality and social cognition, but we do not expect a vague meaning of this term to be widespread in various domains.

**FIGURE 10:**
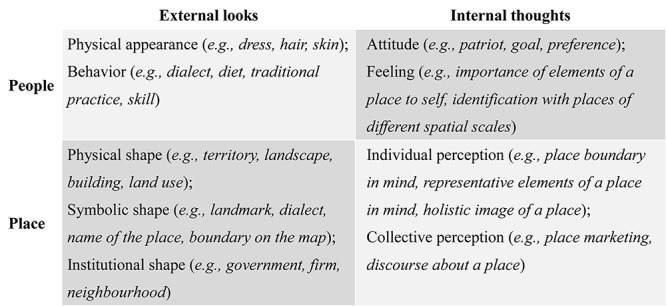
Quadrantal dimensions of the meanings of place identity.

Place identity is more than spatial consciousness. Place identity is claimed as a social construction based on physical reality, but we cannot overlook the objective components and qualities of places and people, which also form part of their identities. We can easily tell a mountain from a lake not because we ascribe high altitude to the mountain or water to the lake but because of the reality that a mountain is high and a lake has water. To explain the meanings of place identity, Paasi (2002c, p.38) draws a diagram showing the connections of the dimensions of place identity. Inspired by Paasi’s work, the social and personal identity theories, and other attempts to identify contents and dimensions of place-related identity (Nario-Redmond et al., 2004; Stobbelaar and Pedrol, 2011; Ramos et al., 2016), we summarized four major dimensions of the meanings of place identity (see Figure 10).

To study people’s place identity or the place identity of a place, the literature generally analyzes them through two perspectives: external looks or internal thoughts. From external looks, researchers observe people’s physical appearances and behaviors associated with a place. How a group of people looks and behaves is thought to be a direct indicator separating this group from others. People’s dress, hair, skin, dialect, diet, and other behaviors are inherited to a great extent. In other words, these characteristics lean on local traditions and genetic inheritance, which have roots in a place. People who have migrated to a new place would keep these origins for a long time. Therefore, the external looks of these characteristics constitute part of people’s public self-identity imprinted by the place where they or their ancestors come from.

As to the external looks of a place, we tend to locate the place on a map at the very beginning. The boundary on the map offers a concrete geographic position and extension of the place for people to identify it in the world. Other tangible elements of a place, such as place names, buildings, land use, population, landscape, landmarks, governments, culture, organizations, and so on, define the external looks of this place together. According to Paasi’s (1986) view on the formation of a region, these elements can be generally grouped into three categories, which are known as physical shape, symbolic shape, and institutional shape. In instructions of a place, one or more of these tangible elements are usually narrated as components that make up the identities of that place.

From the perspective of internal thoughts, researchers strive to design methods to explore subjective connections between people and places. When scientists carry out empirical studies from this view, there is a subtle difference between the methods to measure people’s place identity and the place identity of a place. Respondents are usually asked how they perceive a given place or places in both cases. However, in the case of studying people’s place identity, researchers’ aim emphasizes especially on the meanings and importance of the place(s) people feel in their lives. To attain that, investigations revolve mainly around questions about people’s attitudes and feelings related to a place. Such attitudes are usually value-oriented, such as love for a country, and landscape preferences, which indicate the role of a place in the formation of a belief or standards that guide people’s choices. The feelings can be considered as affect-oriented, which indicates the internalized manifestations of the effects of a place (or elements of a place), such as the importance of elements of a place to the self, and the identification with places of different spatial scales. It is necessary to point out that people living outside of a place are seldom included in this case, because those who have no contacts with the place can hardly perceive the importance of the place in shaping their personality. People’s internal thoughts that reveal their bonds with a place constitute part of their private self-identity imprinted by the place.

With respect to internal thoughts about the place identity of a place, researchers’ major efforts focus on the images of a place people hold in their minds. Such images can be perceived by people living either inside or outside of a place (Paasi, 1986, 2002c). In academic studies and spatial policy-making practices, both collective and individual perceptions of place identities have been paid much attention. Individuals seldom encounter official boundary lines or marks of places in daily life. They often perceive the existence and extent of a place in mind according to the elements of the external looks of the place. From this point, researchers examine the boundary of a place in individuals’ minds to study the intensity of the perceptions of place identity. Besides, individuals bear in mind different representative elements and holistic images of a place, which forms the competitive nature of place identities and becomes the central research interest in this domain. The elements and images of a place in leading social actors’ minds are always shown in place marketing or planning media, discourses, and narratives, with the intention to present to the world what the identity of the place is. In some occasions, the place identity constructed by leading social actors may not be consistent with the place identity perceived by the public, but it may be gradually accepted as the collective place identity through spatial planning, place development, or promotion.

## Conclusion

On the base of the bibliographic records in the core data set of Web of Science related to place identity, this paper identified active research topics and new developments in place identity with a scientometric analysis. An extended survey on measuring methods and roles of place identity in the contents of academic articles was done to supplement the scientometric approach. Distinctions and interrelations between the two meanings of place identity were highlighted with respect to intellectual base, thematic concentration, measurement, and function. Although this paper tried to separate place identity of a place and people’s place identity in multi respects, there are a lot of subtle similarities between their attributes. Both of them are contested, and both are attached with significance in place development, planning, and social conflicts. Similarities also exit in the methods to study them. The establishment or intensity of the identity of a place is often examined by asking about people’s identification with the place. Such similarities could be greater if additional efforts are made to look into the cross-cited articles between the clusters generated by our scientometric analysis. Longtime ignorance of the mixed uses of the two meanings in the literature, in addition to their confusing relationships with other environmental psychological concepts, such as place attachment, has led to a lot of criticism, which can also be blamed for the slow progress in the development of place identity theory.

The quadrantal dimensions of the meanings of place identity presented in this paper are expected to help scholars find their positions when they are involved in research questions in this domain. Place and people are interdependent. Although people’s place identity and place identity of a place are not the same, we did not mean to stress their differences and encourage separated studies on either of them. Instead, places, people, and processes through which both identities are formed should be taken in a comprehensive structure that is supported by a set of theory-grounded principles (Lewicka, 2011). The existing theories relevant to place identity, as well as their interrelations, within the top subject categories identified by our scientometric analysis are not reviewed in this paper, but they are worthy of future study.

To make places better in the contemporary world with increasing mobility and globalization, place identity has been gradually used as an instrument in various domains, such as spatial planning, place marketing, and so forth. However, there are still many outstanding key issues in this area. What should be and how to establish the optimal identity of a specific place? How to reconcile the optimal identity of a place with residents’ and other stakeholders’ place identities? How to measure the costs and benefits in terms of social capital, regional or community resilience, and other human well-being or place growth-related scales, of the transitional process of place identity? More empirical studies are needed to figure out practical solutions and principles for these issues. Otherwise, the research of place identity will always float in the air, so that policy makers and relevant practitioners are not able to touch and operate this tool in practice. Before answering the above questions, we suggest the most fundamental task at present should be to straighten out the differences between place identity and other concepts defining people’s emotional bonds with places and to sort out the basic framework of place identity theory and its relationship with other relevant theories.

## Author Contributions

JP collected and analyzed the data in the manuscript. JP wrote the manuscript. DS introduced the idea and guided the revision of the manuscript. QW offered suggestions for the revision of the manuscript.

## Conflict of Interest

The authors declare that the research was conducted in the absence of any commercial or financial relationships that could be construed as a potential conflict of interest.

## Footnotes

1 The (suggestive) scale of sense of place consists of: (0) not having any sense of place, (1) knowledge of being located in a place, (2) belonging to a place, (3) attachment to a place, (4) identifying with the place goals, (5) involvement in a place, (6) sacrifice for a place.

2 Haartsen et al. (2000) argued that regional identities are not (“natural” or “objective”) characteristics of this area, which is not completely consistent with Paasi’s (1986, 2002c) view. Paasi believed that the “identity of a region” includes “objective scientific classifications” as well.

3 http://project.carrot2.org/index.html

## References

[B1] Amin A. (2004). Regions unbound: towards a new politics of place. *Geografiska Annaler.* 86 33–44. 10.1111/j.0435-3684.2004.00152.x

[B2] Antonsich M. (2010). Meanings of place and aspects of the self: an interdisciplinary and empirical account. *GeoJ.* 75 119–132. 10.1007/s10708-009-9290-9

[B3] Basso K. (1996). *Wisdom Sits in Places: Landscape and Language Among the Western Apache.* New Mexico: University of New Mexico Press.

[B4] Billig M. (1995). *Banal Nationalism.* London: Sage Publications.

[B5] Bleam M. R. (2018). Unbounded place meanings and embodied place identities for conservation volunteers in Scottsdale, Arizona. *J. Environ. Psychol.* 56 76–83. 10.1016/j.jenvp.2018.03.002

[B6] Boland P. (2010). Sonic geography, place and race in the formation of local identity: liverpool and scousers. *Geografiska Annaler.* 92 1–22. 10.1111/j.1468-0467.2010.00330.x

[B7] Bonaiuto M. Breakwell G. M. Cano I. (1996). Identity processes and environmental threat: the effects of nationalism and local identity upon perception of beach pollution. *J. Commun. Appl. Soc. Psychol.* 6 157–175. 10.1002/(sici)1099-1298(199608)6:3<157::aid-casp367>3.0.co;2-w

[B8] Bonaiuto M. Carrus G. Martorella H. Bonnes M. (2002). Local identity processes and environmental attitudes in land use changes: the case of natural protected areas. *J. Econ. Psychol.* 23 631–653. 10.1016/S0167-4870(02)00121-6

[B9] Brace C. (1999). Finding England everywhere: regional identity and the construction of national identity, 1890-1940. *Ecumene* 6 90–109. 10.1177/096746089900600105

[B10] Bradley Q. (2017). Neighbourhood planning and the impact of place identity on housing development in England. *Plann. Theory Pract.* 18 233–248. 10.1080/14649357.2017.1297478

[B11] Bridger J. C. (1996). Community imagery and the built environment. *Sociol. Quart.* 37 353–374. 10.2307/4121288

[B12] Brown B. Perkins D. D. Brown G. (2003). Place attachment in a revitalizing neighborhood: individual and block levels of analysis. *J. Environ. Psychol.* 23 259–271. 10.1016/S0272-4944(02)00117-2

[B13] Buijs A. E. Elands B. H. M. Langers F. (2009). No wilderness for immigrants: cultural differences in images of nature and landscape preferences. *Landsc. Urban Plann.* 91 113–123. 10.1016/j.landurbplan.2008.12.003

[B14] Carrus G. Bonaiuto M. Bonnes M. (2005). Environmental concern, regional identity, and support for protected areas in Italy. *Environ. Behavior.* 37 237–257. 10.1177/0013916504269644

[B15] Chen C. Dubin R. Kim M. C. (2014a). Emerging trends and new developments in regenerative medicine: a scientometric update (2000 – 2014). *Expert Opin. Biol. Therapy* 14 1295–1317. 10.1517/14712598.2014.920813 25077605

[B16] Chen C. Dubin R. Kim M. C. (2014b). Orphan drugs and rare diseases: a scientometric review (2000 – 2014). *Expert Opin. Orphan Drugs* 2 709–724. 10.1517/21678707.2014.920251

[B17] Chow K. Healey M. (2008). Place attachment and place identity: first-year undergraduates making the transition from home to university. *J. Environ. Psychol.* 28 362–372. 10.1016/j.jenvp.2008.02.011

[B18] Cresswell T. (2004). *Place: A Short Introduction.* Oxford: Wiley-Blackwell.

[B19] Devine-Wright P. Lyons E. (1997). Remembering pasts and representing places: the construction of national identities in Ireland. *J. Environ. Psychol.* 17 33–45. 10.1006/jevp.1996.0037

[B20] Devine-Wright P. (2009). Rethinking NIMBYism: the role of place attachment and place identity in explaining place-protective action. *J. Commun. Appl. Soc. Psychol.* 19 426–441. 10.1002/casp.1004

[B21] Devine-Wright P. Clayton S. (2010). Introduction to the special issue: place, identity and environmental behaviour. *J. Environ. Psychol.* 30 267–270. 10.1016/S0272-4944(10)00078-2

[B22] Dixon J. Durrheim K. (2000). Displacing place-identity: a discursive approach to locating self and other. *Br. J. Soc. Psychol.* 39 (Pt 1), 27–44. 10.1348/014466600164318 10774526

[B23] Dredge D. Jenkins J. (2003). Destination place identity and regional tourism policy. *Tourism Geograph.* 5 383–407. 10.1080/1461668032000129137

[B24] Entrikin J. N. (1991). *The Betweenness of Place: Towards A Geography of Modernity.* Baltimore: Johns Hopkins University Press.

[B25] Forester J. (1987). Planning in the face of conflict: negotiation and mediation strategies in local land use regulation. *J. Am. Plann. Assoc.* 53 303–314. 10.1080/01944368708976450

[B26] Fresque-Baxter J. A. Armitage D. (2012). Place identity and climate change adaptation: a synthesis and framework for understanding. *Wiley Interdiscipl. Rev. Clim. Change.* 3 251–266. 10.1002/wcc.164

[B27] Gieseking J. J. Mangold W. Katz C. Low S. Saegert S. (2014). *The People, Place, and Space Reader.* New York, NY: Routledge, Taylor & Francis Group.

[B28] Giuliani M. V. Feldman R. (1993). Place attachment in a developmental and cultural context. *J. Environ. Psychol.* 13 267–274. 10.1016/S0272-4944(05)80179-3

[B29] Gospodini A. (2004). Urban morphology and place identity in European cities: built heritage and innovative design. *J. Urban Design* 9 225–248. 10.1080/1357480042000227834

[B30] Groote P. Haartsen T. (2008). “The communication of heritage: creating place identities” in *The Ashgate Research Companion to Heritage and Identity*, eds Graham B. Howard P. (Hampshire: Ashgate Publishing), 181–194.

[B31] Haartsen T. Groote P. Huigen P. P. P. (2000). *Claiming Rural Identities: Dynamics, Contexts, Policies.* Assen: Van Gorcum.

[B32] Haartsen T. Groote P. Huigen P. P. P. (2003). Measuring age differentials in representations of rurality in The Netherlands. *J. Rural Stud.* 19 245–252. 10.1016/s0743-0167(02)00045-1

[B33] Hague C. Jenkins P. (2005). *Place Identity, Participation and Planning.* Oxfordshire: Routledge.

[B34] Hauge A. L. (2007). Identity and place: a critical comparison of three identity theories. *Architect. Sci. Rev.* 50 44–51. 10.3763/asre.2007.5007

[B35] Hawthorne T. Krygier J. Kwan M. P. (2008). Mapping ambivalence: exploring the geographies of community change and rails-to-trails development using photo-based Q method and PPGIS. *Geoforum* 39 1058–1078. 10.1016/j.geoforum.2007.11.006

[B36] Hernández B. Hidalgo M. C. Salazar-Laplace M. E. Hess S. (2007). Place attachment and place identity in natives and non-natives. *J. Environ. Psychol.* 27 310–319. 10.1016/j.jenvp.2007.06.003

[B37] Hernández B. Martín A. M. Ruiz C. Hidalgo M. D. C. (2010). The role of place identity and place attachment in breaking environmental protection laws. *J. Environ. Psychol.* 30 281–288. 10.1016/j.jenvp.2010.01.009

[B38] Huigen P. P. P. Meijering L. (2005). “Making places: a story of the Venen,” in *Senses of Place, Senses of Time*, eds Ashworth G. J. Graham B. (Burlington: Ashgate), 19–30.

[B39] Hull R. B. Lam M. Vigo G. (1994). Place identity: symbols of self in the urban fabric. *Landsc. Urban Plann.* 28 109–120. 10.1016/0169-2046(94)90001-9

[B40] Hurrell A. (1995). “Regionalism in theoretical perspective,” in *Regionalism in World Politics: Regional Organization and International Order*, eds Fawcett L. Hurrell A. (Oxford: Oxford University Press), 37–73.

[B41] Jorgensen B. S. Stedman R. C. (2001). Sense of place as an attitude: lakeshore owners attitudes toward their properties. *J. Environ. Psychol.* 21 233–248. 10.1006/jevp.2001.0226

[B42] Kacowicz A. M. (1999). Regionalization, globalization, and nationalism: convergent, divergent, or overlapping? *Alternat. Global Local Political.* 24 527–555. 10.1111/1467-9701.00250

[B43] Keegstra R. (2009). *Representations of the Loire Valley.* Master thesis, University of Groningen, Groningen

[B44] Knapp W. (2006). “Planning in peri-urban regions: on regional identity and organizing capacity,” in *Europe’s City-Regions Competitiveness: Growth Regulation and Peri-Urban Land Management*, eds Bertrand N. Kreibich V. (Assen: Uitgeverij Van Gorcum), 61–84.

[B45] Knez I. Butler A. Sang Å. O. Ångman E. Sarlöv-Herlinc I. Åkerskog A. (2018a). Before and after a natural disaster: disruption in emotion component of place-identity and wellbeing. *J. Environ. Psychol.* 55 11–17. 10.1016/j.jenvp.2017.11.002

[B46] Knez I. Sang Å. O. Gunnarsson B. Hedblom M. (2018b). Wellbeing in urban greenery: the role of naturalness and place identity. *Front. Psychol.* 9:491. 10.3389/fpsyg.2018.00491 29695984 PMC5904257

[B47] Kyle G. T. Absher J. D. Graefe A. R. (2003). The moderating role of place attachment on the relationship between attitudes toward fees and spending preferences. *Leisure Sci.* 25 33–50. 10.1080/01490400306552

[B48] Kyle G. Graefe A. Manning R. Bacon J. (2004). Effects of place attachment on users’ perceptions of social and environmental conditions in a natural setting. *J. Environ. Psychol.* 24 213–225. 10.1016/j.jenvp.2003.12.006

[B49] Lalli M. (1992). Urban-related identity: theory, measurement, and empirical findings. *J. Environ. Psychol.* 12 285–303. 10.1016/S0272-4944(05)80078-7

[B50] Lengen C. Kistemann T. (2012). Sense of place and place identity: review of neuroscientific evidence. *Health Place* 18 1162–1171. 10.1016/j.healthplace.2012.01.012 22440778

[B51] Lewicka M. (2008). Place attachment, place identity, and place memory: restoring the forgotten city past. *J. Environ. Psychol.* 28 209–231. 10.1016/j.jenvp.2008.02.001

[B52] Lewicka M. (2010). What makes neighborhood different from home and city? Effects of place scale on place attachment. *J. Environ. Psychol.* 30 35–51. 10.1016/j.jenvp.2009.05.004

[B53] Lewicka M. (2011). Place attachment: how far have we come in the last 40 years? *J. Environ. Psychol.* 31 207–230. 10.1016/j.jenvp.2010.10.001

[B54] Low S. M. Altman I. (1992). *Place Attachment: A Conceptual Inquiry.* New York, NY: Plenum.

[B55] Manzo L. C. Perkins D. D. (2006). Finding common ground: the importance of place attachment to community participation and planning. *J. Plann. Lit.* 20 335–350. 10.1177/0885412205286160

[B56] Massey D. (1994). *Space, Place and Gender.* Cambridge: Polity.

[B57] Massey D. (1995). “The conceptualization of place,” in *A Place in the World*, eds Massey D. Jess P. (Oxford: Oxford University Press), 45–85.

[B58] Melnychuk A. Gnatiuk O. (2018). Regional identity and the renewal of spatial administrative structures: the case of Podolia, Ukraine. *Nephron Clin. Pract.* 26 42–54. 10.2478/mgr-2018-0004

[B59] Miloslav Š (2018). The regional identity of the inhabitants of regions which have experienced an interrupted continuity in their socio-historical development. A case study of Czech regions that were resettled after World War II. *Geografie* 123 437–459. 10.37040/geografie2018123040437

[B60] Nario-Redmond M. R. Biernat M. Eidelman S. Palenske D. J. (2004). The social and personal identities scale: a measure of the differential importance ascribed to social and personal self-categorizations. *Self Identity* 3 143–175. 10.1080/13576500342000103 21218374

[B61] Nogué J. Vicente J. (2004). Landscape and national identity in Catalonia. *Political Geogr.* 23 113–132. 10.1016/j.polgeo.2003.09.005

[B62] Paasi A. (1986). The institutionalization of regions: a theoretical framework for the understanding of the emergence of regions and the constitution of regional identity. *Fennia* 164 105–146. 10.11143/9052

[B63] Paasi A. (1991). Deconstructing regions: notes on the scales of spatial life. *Environ. Plann. A* 23 239–256. 10.1068/a230239

[B64] Paasi A. (1996). *Territories, Boundaries and Consciousness.* Chichester: Wiley.

[B65] Paasi A. (2001). Europe as a social process and discourse: considerations of place, boundaries and identity. *Eur. Urban Regional Stud.* 8 7–28. 10.1177/096977640100800102

[B66] Paasi A. (2002a). Bounded spaces in the mobile word: deconstructing ‘regional identity’. *Tijdschrift Voor Econ. Soc. Geografie* 93 137–148. 10.1111/1467-9663.00190

[B67] Paasi A. (2002b). Place and region: regional worlds and words. *Progr. Hum. Geogr.* 26 802–811. 10.1191/0309132502ph404pr

[B68] Paasi A. (2002c). “Regional identities and the challenge of the mobile world,” in *Kulturell Identitet og Regional Utvikling*, ed. Engen T. O. (Elverum: Høgskolen i Hedmark), 33–48.

[B69] Paasi A. (2003). Region and place: regional identity in question. *Progr. Hum. Geogr.* 27 475–485. 10.1191/0309132503ph439pr

[B70] Paasi A. (2004). Place and region: looking through the prism of scale. *Progr. Hum. Geogr.* 28 536–546. 10.1191/0309132504ph502pr

[B71] Paasi A. (2009a). “Regions and regional dynamics,” in *The SAGE Handbook of European Studies*, ed. Rumford C. (Los Angeles, CA: SAGE), 464–484. 10.4135/9780857021045.n26

[B72] Paasi A. (2009b). The resurgence of the ‘region’ and ‘regional identity’: theoretical perspectives and empirical observations on regional dynamics in Europe. *Rev. Int. Stud.* 35 121–146. 10.1017/S0260210509008456

[B73] Peterson G. (1988). Local symbols and place identity: tucson and albuquerque. *Soc. Sci. J.* 25 451–461. 10.1016/0362-3319(88)90024-9

[B74] Pinto C. T. (2000). “Landscape identity, a key for integration,” in *Lebenscraum Landschaft*, ed. Pedroli B. (Rotterdam: Uitgeverij Christofoor), 145–149.

[B75] Pretty G. H. Chipuer H. M. Bramston P. (2003). Sense of place amongst adolescents and adults in two rural Australian towns: the discriminating features of place attachment, sense of community and place dependence in relation to place identity. *J. Environ. Psychol.* 23 273–287. 10.1016/S0272-4944(02)00079-8

[B76] Proshansky H. M. (1976). *Environmental Psychology: People and Their Physical Setting.* New York, NY: Holt, Rinehart and Winston.

[B77] Proshansky H. M. (1978). The city and self-identity. *Environ. Behav.* 10 147–169. 10.1177/0013916578102002

[B78] Proshansky H. M. Fabian A. K. (1987). “The development of place identity in the child,” in *The Built Environment and Child Development*, eds Weinstein C. S. David T. G. (New York, NY: Plenum Press), 21–40.

[B79] Proshansky H. M. Fabian A. K. Kaminoff R. (1983). Place-identity: physical world socialization of the self. *J. Environ. Psychol.* 3 57–83. 10.1016/S0272-4944(83)80021-8

[B80] Raagmaa G. (2002). Regional identity in regional development and planning. *Eur. Plann. Stud.* 10 55–76. 10.1080/09654310120099263

[B81] Relph E. (1976). *Place and Placelessness.* London: Pion.

[B82] Rijnks R. H. Strijker D. (2013). Spatial effects on the image and identity of a rural area. *J. Environ. Psychol.* 36 103–111. 10.1016/j.jenvp.2013.07.008

[B83] Ritalahti J. (2008). “Regional identity in destination development”, in *Proceedings of the Conference on Regional Development and Innovation Processes, 5-7 March*, Porvoo-Borga.

[B84] Rollero C. Piccoli N. D. (2010). Does place attachment affect social well-being? *Eur. Rev. Appl. Psychol.* 60 233–238. 10.1016/j.erap.2010.05.001

[B85] Ramos I. L. Bernardo F. Ribeiro S. C. Van Eetvelde V. (2016). Landscape identity: implications for policy making. *Land Use Policy* 53 36–43. 10.1016/j.landusepol.2015.01.030

[B86] Saleh M. A. E. (1998). Place identity: the visual image of Saudi Arabian cities. *Habitat Int.* 22 149–164. 10.1016/S0197-3975(97)00033-7

[B87] Scannell L. Gifford R. (2010). Defining place attachment: a tripartite organizing framework. *J. Environ. Psychol.* 30 1–10. 10.1016/j.jenvp.2009.09.006

[B88] Semian M. Chromy P. (2014). Regional identity as a driver or a barrier in the process of regional development: a comparison of selected European experience. *Norsk Geografisk Tidsskrift.* 68 263–270. 10.1080/00291951.2014.961540

[B89] Shamai S. (1991). Sense of place: an empirical measurement. *Geoforum* 22 347–358. 10.1016/0016-7185(91)90017-K

[B90] Shi Y. Liu X. (2019). Research on the literature of green building based on the Web of Science: a scientometric analysis in CiteSpace (2002–2018). *Sustainability* 11:3716 10.3390/su11133716

[B91] Simon C. Huigen P. Groote P. (2010). Analysing regional identities in the Netherlands. *Tijdschrift Voor Econ. Soc. Geografie* 101 409–421. 10.1111/j.1467-9663.2009.00564.x

[B92] Stedman R. C. (2002). Toward a social psychology of place: predicting behavior from place-based cognitions, attitude, and identity. *Environ. Behav.* 34 561–581. 10.1177/0013916502034005001

[B93] Stewart W. P. Liebert D. Larkin K. W. (2004). Community identities as visions for landscape change. *Landsc. Urban Plann.* 69 315–334. 10.1016/j.landurbplan.2003.07.005

[B94] Stobbelaar D. J. Pedrol B. (2011). Perspectives on landscape identity: a conceptual challenge. *Landsc. Res.* 36 321–339. 10.1080/01426397.2011.564860

[B95] Stokols D. Shumaker S. A. (1981). “People in places,” in *Cognition, Social Behavior, and the Environment*, ed. Harvey J. H. (Hillsdale, NJ: Lawrence Erlbaum Associates), 441–488.

[B96] Stoll-Kleemann S. (2001). Barriers to nature conservation in Germany: a model explaining opposition to protected areas. *J. Environ. Psychol.* 21 369–385. 10.1006/jevp.2001.0228

[B97] Terada T. (2003). Constructing an ‘East Asian’ concept and growing regional identity: from EAEC to ASEAN+3. *Pacific Rev.* 16 251–277. 10.1080/0951274032000069615

[B98] Terluin I. J. (2003). Differences in economic development in rural regions of advanced countries: an overview and critical analysis of theories. *J. Rural Stud.* 19 327–344. 10.1016/S0743-0167(02)00071-2

[B99] Tuan Y. F. (1977). *Space and Place: The perspective of experience.* Minneapolis, MN: University of Minnesota Press.

[B100] Twigger-Ross C. L. Bonaiuto M. Breakwell G. (2003). “Identity theories and environmental psychology,” in *Psychological Theories for Environmental Issues*, eds Bonnes M. Lee T. Bonaiuto M. (Farnham: Ashgate Publishing Limited), 203–233.

[B101] Urquhart J. Acott T. (2013). Constructing ‘The Stade’: fishers’ and non-fishers’ identity and place attachment in Hastings, south-east England. *Mar. Policy* 37 45–54. 10.1016/j.marpol.2012.04.004

[B102] Uzzell D. Pol E. Badenas D. (2002). Place identification, social cohesion, and enviornmental sustainability. *Environ. Behav.* 34 26–53. 10.1177/0013916502034001003

[B103] Vainikka J. (2012). Narrative claims on regions: prospecting for spatial identities among social movements in Finland. *Soc. Cult. Geogr.* 13 587–605. 10.1080/14649365.2012.710912

[B104] Van den Berg A. E. (1999). *Individual Differences in the Aesthetic Evaluation of Natural Landscapes.* PhD dissertation of the University of Groningen, Groningen.

[B105] Van Houtum H. Lagendijk A. (2001). Contextualising regional identity and imagination in the construction of polycentric urban regions: the cases of the Ruhr area and the Basque country. *Urban Stud.* 38 747–767. 10.1080/00420980120035321

[B106] Van Rekom J. Go F. (2006). Being discovered: a blessing to local identities? *Ann. Tourism Res.* 33 767–784. 10.1016/j.annals.2006.03.002

[B107] Vidal T. Berroeta H. de Masso A. Valera S. Peró M. (2013). Place attachment, place identity, sense of community, and local civic participation in an urban renewal context. *Estudios Psicol.* 34 275–286. 10.1174/021093913808295172

[B108] Wheeler R. (2015). Reconciling windfarms with rural place identity: exploring residents’ attitudes to existing sites. *Sociol. Ruralis* 57 110–132. 10.1111/soru.12121

[B109] White D. D. Virden R. J. Riper C. J. V. (2008). Effects of place identity, place dependence, and experience-use history on perceptions of recreation impacts in a natural setting. *Environ. Manage.* 42 647–657. 10.1007/s00267-008-9143-1 18535854

[B110] Williams D. R. Roggenbuck J. W. (1989). “Measuring place attachment: some preliminary results”, in *Proceedins of the Abstracts of the 1989 Leisure Research Symposium*, eds McAvoy L. H. Howard D. (Arlington, VA: National Recreation and Park Association), 32.

[B111] Xu M. de Bakker M. Strijker D. Wu H. (2015). Effects of distance from home to campus on undergraduate place attachment and university experience in China. *J. Environ. Psychol.* 43 95–104. 10.1016/j.jenvp.2015.05.013

[B112] Yuen B. (2005). Searching for place identity in Singapore. *Habitat Int.* 29 197–214. 10.1016/j.habitatint.2003.07.002

[B113] Zimmerbauer K. (2011). From image to identity: building regions by place promotion. *Eur. Plann. Stud.* 19 243–260. 10.1080/09654313.2011.532667

[B114] Zimmerbauer K. Suutari T. Saartenoja A. (2012). Resistance to the deinstitutionalization of a region: borders, identity and activism in a municipality merger. *Geoforum* 43 1065–1075. 10.1016/j.geoforum.2012.06.009

